# On the Present State of Medicine in Denmark

**Published:** 1837-04

**Authors:** C. Otto

**Affiliations:** Professor of Medicine in the University of Copenhagen


### PART FIFTH.

### Medical Intelligence.

---

### ON THE PRESENT STATE OF MEDICINE IN DENMARK;

By C. OTTO, M.D., Professor of Medicine in the University of Copenhagen.

### SECOND REPORT.

### ON THE PUBLIC HOSPITALS, CHARITIES, &c. IN COPENHAGEN.

THE capital of Denmark, partly through the liberality of the government, and partly through the charity of private individuals, is so well provided with institutions for the support and relief of the poor and the sick, that it may with justice be said to equal, and even excel, most other large cities in this respect.

#### 1. THE ROYAL FREDERICK'S HOSPITAL.

This hospital, which owes its origin to the beneficence of King Frederick the Fifth, is situated in an airy part of the town, near the harbour. It was founded in the year 1751, but not completed until 1757. It is supported by different endowments, made at different times by opulent private citizens, and its capital at the present moment amounts to about 100,000*l*. The buildings of the hospital form a large square, including a court, with the fronts on two sides facing two different streets. To the place and manner in which it has been built, as well as to many of the arrangements, certainly much may be objected. It is situated in a low and humid locality, and the court between the buildings is too small, so that the air can have no free access; an evil which is further increased by trees, with which the space is filled. The two sides of the square in which the wards are, have only one story, and even this is a ground floor, without any cellars below. The wards themselves are of different sizes; some being no less than thirty-seven and three-quarters yards in length, and nine yards broad; others are only one-third part so long; of all, the height is six yards. Most of the wards are so badly arranged, that the only entrance into them is through other wards. There are no particular rooms for convalescents, except for the fever patients; no particular refectories; no rooms where the patients are received, nor places for drying quickly the linen of the patients. The beds (which are of wood) are of different sizes, without curtains, but provided with moveable screens.

Notwithstanding these and other faults, when we consider the remote period at which the hospital was built, it must, upon the whole, be considered a good one, and it is certainly well managed. As to cleanliness, ventilation, &c., nothing can now be objected to it.

The hospital has a separate building for warm, cold, shower, and Russian vapour baths; a very good dissecting room, and an excellent operating room. In both the side buildings, turning into the yard, are the wards for the poorer patients; on the one side the medical, on the other the surgical. The fever wards are in the middle buildings; and particular rooms for single patients, who can pay for their accommodation, are distributed in those parts of the buildings that front the streets.

According to the charter granted to the hospital in 1756, it was destined to receive poor and indigent patients who get no support from other benevolent institutions, and whose diseases are not deemed incurable; but, according to a later rescript, in 1772, now also such patients are admitted who are able to pay; the fine paid being different according to the bed, the diet, &c. It is, however, never sufficient to indemnify the institution: it is usually six shillings per week. Incurable patients are never admitted; neither are patients received with the venereal disease, the small-pox, insanity, &c., nor children under the age of seven years. The hospital can contain 350 patients, and the average number of sick who receive relief in it amounts annually to about 3000.

When anybody wishes to be admitted into the hospital, he must be announced at the office of the inspector of the institution, where he either is to deliver a certificate of being indigent, from one of the overseers of the poor, or a signed verification from some other known and trustworthy person, that the requisite money will be paid for the maintenance of the patient in the hospital. The candidate is then visited the same day or the next, according to his disease, at his home, by one of the medical or surgical assistants of the hospital; and, if he is not affected with any disease that excludes him, he is immediately received, if there be a vacant bed.

The medical officers are as follows:—1. The chief physician, (at present Dr. O. Bang,) professor of therapeutics at the university.) 2. The chief surgeon, (at present Mr. G. Möller, adjunct at the College of Surgeons.) They are only appointed for six years, receive a royal salary of 800 dollars (80*l.*) and have free lodging in the hospital; their period of office may nevertheless be protracted, and it commonly is so. 3. A physician of reserve. 4. A surgeon of reserve, who is chosen by turns out of the young lecturers at the College of Surgeons. These two last-mentioned officers only keep their office during two years. 5. Four medical, and, 6. Four surgical assistants, (called "candidates,") who also lodge in the hospital, and are appointed for four years. An apothe cary also lives in the hospital.

All students are allowed to enter their names with the chief physician or the surgeon as "volontiers;" and, as such, not only attend his visits at the wards, but take often an active part in the treatment of the patients, and in the keeping of the journals, under the superintendence of the physician or the surgeon. Every other student, however, has a free admission to the patients during the public visits of the medical officers, which are every morning between seven and eight, and every afternoon between four and five. Most of the students obtain in this manner their practical education at this hospital, without even the least expense to them. Journals of the history and treatment of the diseases are carefully kept *in duplo;* and the bodies of those dying in the hospitals are for the most part examined, the permission of the relations being always obtained, as no prejudice against such examinations exists amongst the Danish people. The annual mortality of the hospital is one in eleven or twelve.

With this hospital a medical and surgical clinic are combined. The medical, by Professor Bang, (every Wednesday and Saturday, from three to four,) is very instructive. One of the students, or the professor himself, examines the patient who has been admitted, and remarks are then made upon the disease. Every course of the clinic begins with a clinical lecture. The surgical clinic, by Professor Möller, (Monday, Wednesday, and Saturday, from nine to ten,) is also very instructive.

### II. THE GENERAL HOSPITAL, ("*Almindelig Hospital.*")

This hospital is situated in an airy part of the town, in the neighbourhood of the Frederick's Hospital, and is supported from the poor-fund and by legacies. It is not only an hospital for patients, but also an almshouse. The patients in the Hospital are of course separated from the members of the Almshouse. The number of beds for the sick is now four hundred, and all such poor patients who cannot be admitted into the Frederick's Hospital on account of their disease are received here; and also many who can pay for their maintenance. The manner of announcing, visiting, and admitting the patients, is the same as at the Frederick's Hospital.

The wards are twenty-three in number, the largest with eighteen beds, some with sixteen, twelve, ten, some with only six; they are much better arranged here than in the other hospital. They are distributed in the upper stories, of which there are four, and all open on lobbies; so that no ward is in a direct communication with another. The wards themselves, furnished with a water-closet, are spacious, very high, and excellently ventilated; the beds (here also of wood,) have a sufficient space between them, and are without curtains, but provided with moveable screens. There are particular fever-wards, and particular wards for itch-patients and for contagious diseases. There are separate rooms for women affected with syphilis. There are airy courts for the exercise of convalescents, and cold and warm sulphur baths. There are also ovens, in which the clothes of patients infected with contagious diseases are cleansed. For the many patients attacked by cutaneous diseases who seek relief in the hospital, there is a floating raft of timber, with covered cells, on the sea behind the hospital, where sea-baths daily are administered in the summer time.

More than two thousand patients are annually admitted into this hospital, and the mortality is from seven to eight in the hundred. The medicines for the wards are supplied from a dispensatory in the hospital itself, which likewise furnishes all the poor in the town with medicines and treatment gratuitously. The great regard that is paid to the comfort and conveniences of the sick poor admitted into this hospital is proved by the following notice, printed in the Danish, German, French, and English languages, and hung up in all the wards: "The chief physician, who visits the wards daily, begs to be informed, when on his visits, not only of the state of the patients, but likewise, *without restraint*, of every thing the patients deem necessary to relieve their sufferings, or render their condition more comfortable. They are also to inform him if any deficiency is experienced in regard to nourishment, cleanliness, attendance, or care. Notice will be taken of every complaint that is sufficiently grounded, and the fault or faults will be instantly rectified."

The officers of this hospital are the following: 1. A chief physician, (Prof. Wendt, who also is staff-surgeon to the Danish army.) 2. A chief surgeon, (Prof. R. Thal.) 3. A physician of reserve. 4. A surgeon of reserve; and 5. Four medical and four surgical assistants, called "candidates." To Prof. Wendt the hospital owes many obligations for his improvements in the manner of ventilating the wards, for many new accommodations for the patients, &c. He continues to labour zealously in his office, and, having made it a rule to open every dead body, he will, on some future occasion, be able to lay before the medical public a valuable collection of pathological facts. Three years ago he published a good description of the hospital, in Danish, with plates.

The Almshouse in this institution consists of twelve rooms; two of which are for twenty, the others for twenty-four persons each. At the present moment there are about eight hundred indigent and old persons in the house, of whom 250 are males and 550 females. Every one has his own bed and a sufficiently large repository for his small comforts. The weakest individuals lodge in the lower stories, that they may more conveniently get into the court to enjoy the fresh air. They get a dinner and a pound of bread daily; and the payment for what work they are able to perform comes to them. Nevertheless they are obliged to do what is wanted in the hospital; as, for instance, to wash the linen, to cleanse the staircases and rooms, &c. There are rooms set apart for spinning, and one for picking oakum. The inmates of the Almshouse are only allowed to walk into the town on Sundays and other holidays.

### III. THE NAVAL HOSPITAL.

This is a very fine and comfortable building, opened in the year 1806, and situated in "Nyboder," or that part of the town where all the inferior naval officers and sailors live. This hospital affords medical and surgical assistance only to the sailors and the workmen and mechanics belonging to the royal dock-yard, and to some of the wives and the children of the sailors of the royal navy. From thirteen to sixteen hundred patients are annually treated here. As all the sailors are divided into two divisions, there are also two surgeons for them, Dr. Winckler and Dr. Mansa. Each treats the patients of his own division in the hospital, and each has a surgeon of reserve. Those sick sailors who suffer from a slight disease, or cannot be received into the hospital, are treated in their own houses, free of expense, by two other surgeons of reserve.

IV. THE LAZARETTO OF THE NAVY, *(Söquesthuset.)*

This establishment is independent of the last, although belonging to the navy. It is situated in a different part of the town, (Christianshaven,) and is a fine spacious building. It was founded in the reign of Frederick the Third. It is exclusively appropriated for wounded and maimed sailors during war. There is sufficient space for one thousand beds, and the whole medical and surgical stores, which fortunately escaped the rapacious hands of the English in 1807, are kept here. At the present time it is not used as a hospital. During the late epidemics of the small-pox, and while quarantine was observed, all the patients affected with it were brought here. A part of the building is now used as a prison.

V. THE MILITARY HOSPITAL.

This is situated in an isolated and airy part of the town, and is appropriated as an asylum for sick and wounded officers and soldiers in the Danish army, and for the sick wives and children of the soldiers. Of officers, only lieutenants and staff-captains are received. Over the entrance is a Danish inscription, as follows: "Frederick the Sixth, in the year 1817, caused this building, which was erected by his royal ancestor, to be converted into a military hospital." A rich Jew, Mr. A. Meyer, bequeathed about three thousand pounds to this hospital. From four to five thousand patients are annually treated in it, and the men of every regiment by their own army-surgeons. The wards are spacious and well ventilated; the court-yard very large; and the whole establishment is excellently arranged for the comfort and convenience of the officers.

VI. THE HOSPITAL OF THE CIVIL PENITENTIARY.

This establishment consists of a particular building in the House of Correction. In the arrangement of the wards, of which there are eight, and the other conveniences commonly found in hospitals, this hospital is not inferior to any one. About one thousand patients are treated here annually. The writer of this Report is physician to the hospital, and intends in another article, destined for this Journal, to enlarge upon the diseases of prisoners in general, and of the Danish ones in particular; and he then will have a better opportunity of making the readers acquainted with this hospital.

VII. THE LYING-IN HOSPITAL.

The Danish nation is greatly indebted to King Frederick the Fifth, who founded this hospital in 1750, which really is one of the most celebrated hospitals of this sort in Europe. It is situated in the same street as the General Hospital, and opposite to it. It is supported by royal beneficence, and by several considerable bequests, particularly of one of the queen-dowager, Juliane Maria. The hospital is appropriated to three uses: 1, to receive unmarried and also a certain number of married women, either gratuitously or for payment; 2, to instruct midwives and accoucheurs in practical midwifery; 3, to provide for all the children of unmarried people, when the mothers either will not or cannot themselves take care of them.

The number of those who are born in the hospital is annually about one thousand, being a third part of the whole births in the town! The lying-in women have all possible comforts, and fifty can enjoy them at the same time, although commonly about thirty only are to be found there at once. Those who do not pay for their maintenance while in the house, are as one to five of those who pay. In order to prevent infanticides, all unmarried pregnant women, when they are about to be delivered, are bound to seek admission into the hospital.

This hospital surpasses in utility all other similar institutions in Europe. All women who wish to practise as midwives must be educated here, and thirty-six of such (of which number twenty-four do not pay,) obtain here annually all the necessary practical instruction by a midwife who lives in the hospital, and by the chief physician of it, the celebrated Professor T. S. Saxtorph, who gives lectures to them adapted to their understanding. After the lapse of the year they are examined by Prof. Saxtorph, the physician of reserve, and the town physician; and it is only after having been found sufficiently qualified that they are allowed to practise midwifery in Denmark, and this only under certain restrictions. They are always bound to call in a physician and surgeon, (all such practise midwifery,) whenever a delivery is very difficult, and an operation, manual or instrumental, is required. But the hospital is also appropriated to gratuitous practical education in midwifery, of physicians and surgeons. Four students (two examined at the Medical Faculty and two at the College of Surgeons,) are always studying here practical midwifery during four months, (there are, of consequence, twelve students every year,) during which time they have free lodging in the house, partake in the examinations, and attend at the delivery of all the women, and perform the instrumental operations that are deemed necessary. They are also very often sent for to execute difficult deliveries in the town, (on which occasions two always go together, the eldest and the youngest,) so that they never leave the hospital without having officiated in many difficult deliveries, and performed numerous obstetrical operations. I have not seen, during all my medical travels through Europe, such an excellent school as this for practical midwifery, where the student can get instructed with such facility, and without any expense, in this important branch of our art.

The hospital may also be considered as a foundling hospital, in taking care of the children left by their parents, until they can be brought into another charitable institution appropriated to the education of orphans or friendless children. —The chief physician is Prof. T. S. Saxtorph, and the physician of reserve, Prof. Aschroch, who both have a magnificent lodging in the hospital, free of expense.

### VIII. ST. JOHN'S HOSPITAL.

This hospital, commonly called "Bidstrupgaard," is the only house for lunatics for Zealand and the islands, although there are similar smaller ones in several of the minor towns. It is not in Copenhagen, but situated twenty English miles distant from it, and two miles from Rotschild, near Issetjorden, on the sea-coast. It possesses one of the finest landscapes, and a fine park adorned with woods. We feel it a duty incumbent upon us to give a full description of this hospital, that the medical public abroad may be able to judge of what is done in Denmark for the relief and cure of the insane; a subject which still, in our enlightened times, is very much neglected in many countries.

The St. John's Hospital consists of a main building and two wings, all of stone. The main building, two stories high, and destined partly for such patients as are curable and behave themselves quietly, and partly for convalescents, has two divisions, the one for males, the other for females. The greater number of the wards in this building are large and airy, and fitted to receive three, six, or more patients. Along the whole centre of the building, in both stories, there is a small gallery, on both sides of which the wards open, with a single entrance. In the lower story is the apothecary's shop, and a room for the second physician, who has the day-watch. On the ground-floor lodge the other inferior physician and some watchmen.

The wings of the hospital have only one story; one of the wings is for male, the other for female patients. Along these wings, and towards the garden, there is a large, high, and light lobby, which in the winter is warmed, and during the dark seasons lighted by lamps. In this the patients take their exercise when the weather or the season prevents them from walking out in the court-yard or in the garden. From the lobby the wards are warmed through tubes of iron, commonly one for every two wards. These wards are arranged for several patients; but in one of the wings there are some smaller ones for the unquiet persons, in which the windows are higher up, so that the patients cannot get to them. Ventilation is insured by the opening of these windows, and one opposite to them over the door to the lobby. In these smaller wards there is only one bed, placed in the middle of the room, and furnished with a wooden bottom sloping from the sides to the centre, in which there is an opening for the escape of the urine and fæces, in the case of the patient being very filthy. To this bed the violent patients can be fastened by means of straps. In the corner of these rooms there is a water-closet, fastened into the wall, and in this an aperture, sufficiently large to admit of the close-stool being taken out from without, by which arrangement all offensive effluvia are speedily got rid of.

At the side of each of the wings of the building there is a large room, where a great part of the patients, particularly of the lower classes, are assembled under inspection, and occupy themselves with different handiworks. Every wing has its own court-yard, surrounded by a wooden fence, where the patients, when not at work, may enjoy bodily exercise in the fresh air; and before the main building there is a larger court, where both male and female patients walk about.

It is not allowed to any patient, during the daytime, to stay in the bed-room or to remain in bed, if he does not suffer from any disease that makes it necessary; and, in this last case, there are particular rooms appropriated for those who are sick. Some exception to this rule is nevertheless made in favour of patients of the higher classes, who often get permission to occupy themselves in their own rooms with reading, writing, &c. There are also particular rooms where the patients breakfast, dine, and sup, and rooms for more quiet and orderly patients and for convalescents, where they may entertain themselves with newspapers, books, or games; as, for instance, chess, draughts, &c. In the main building there are some smaller dining-rooms for patients of the higher classes, who are allowed better fare, and dine at a common table.

Both curable and incurable insane are received into this hospital; it is under the administration of the royal board of the poor in Copenhagen, and is visited several times in the year by one or more of its members, for the purpose of seeing that every thing is in proper order.

The hospital is furnished with all sorts of baths, and has a very fine garden and a park. The endowments which it possesses are very considerable, and amount nearly to eighty thousand pounds. On this account many poor persons are admitted without any payment; many pay, on the contrary, for their maintenance while there: those who get a better board pay ten shillings per week, and those who get the usual board six shillings. The daily number of lunatics is 150, and from twenty to forty of the number annually admitted are cured.

The hospital is superintended by a physician and an inspector: the former has under him two other physicians, (of whom, one dispenses the medicines,) and the latter a clerk. They all live in the hospital, and have tolerably good salaries and excellent lodgings. The physician is not allowed to extend his private practice beyond the limits of five English miles from the hospital.

The daily fare of the patients is of four different sorts: 1, the better one for the higher classes; 2, the usual or simple fare; 3, one for those at work, (a greater portion of bread and butcher meat, a luncheon, and a supper;) and, 4, one for the sick. The physician has besides permission to make what changes he deems necessary in any one's diet.

All the patients of the lower classes are clothed in the same manner, whether the clothes are afforded by the individuals themselves or by the institution. The bedclothes consist of two mattresses stuffed with halm, a pillow stuffed with the same, two sheets, and a coverlet of wool or of feathers. The greatest cleanliness and order exist, and the institution is provided with such a considerable store of linen, shirts, &c., that the patients can run no risk of being kept uncleanly.

Whenever a patient is received into the hospital, he is carried into the bath-house, where he is undressed, washed, combed, bathed, and then dressed in clean linen and in the clothes of the hospital. This rule is even observed with the most part of those who bring clothes with them or provide their own, as they are not allowed to wear their own clothes before they have been well examined, to see that nothing by which the patient can do harm to himself has been concealed in them.

There are fixed rules regarding the order in the hospital and the duties which everybody belonging to it has to perform. Every thing is done with the utmost regularity, and at fixed times of the day. The patients, for instance, must rise in the morning, go to work, dine, &c. at a certain fixed hour. It is evident how much such order and regularity, which are commonly wanting in the domestic circle, must influence the cure of the patients.

When a lunatic is admitted, reports of his previous life, of his situation, his previous diseases, of the probable causes of his insanity,—in short, of every circumstance that may elucidate his present state and facilitate the cure is required to be known. There is a particular form, containing certain questions on these points, which always are to be answered by the relations or the former physician of the patient.

In regard to the causes of mental aberration here observed, the physician, Dr. A. Göricke, a very intelligent and well-instructed practitioner, considers the principal to be poverty, dissoluteness of manners, manustupration, &c., and, particularly of late, abuse of spirituous liquors. The most usual forms in which insanity here manifests itself are idiocy, periodical mania, and melancholia. The mental aberration combined with fixed ideas, occurs more seldom. Epilepsy, with or without insanity, seems here less frequent than in the madhouses in other countries; and, when this complication occurs, it is considered the most difficult of cure by Dr. Göricke, as well as by all other physicians of lunatics: in fact, he has seen no instance of its being cured. The physician keeps a very accurate journal of all the cases, in which are noted the name, age, and former residence of the lunatic; the information that has been obtained about the course and the causes of the disease; the observations that are made in the hospital respecting the bodily and mental state of the patient at his reception, the means that are employed against it, its results, &c.

The treatment is of course accommodated to the individual circumstances of every patient, but the principles according to which Dr. G. acts are the following: He endeavours, first of all, to win the confidence and love of the patient by a humane treatment and a soothing manner, united with seriousness. Only in particular cases does he employ force and coercion, and then only where mildness is unable to accomplish the object in view. As means of coercion, Dr. G. almost always uses the common strait-jacket, deeming most others, and especially the coercion-stool of Coxe, of no use, and even not seldom noxious. For this purpose, however, he often employs shower and douche baths. As a usual punishment for the disobedience of orders, Dr. G. diminishes the daily fare, prohibits the walking in the garden or in the lobby in company with the others, or he denies them the use of snuff, &c. The second principle which Dr. G. follows in his treatment of the insane is to separate the patients according to the state of their faculties, former manner of living, &c.; and, although this separation cannot be executed in the most perfect degree, Dr. G. does his utmost in this respect. There is still wanted a separate building, only for convalescents, and this is intended soon to be provided. The third principle in the treatment is to occupy the patients as much as possible with bodily labour and mental recreations, so that they are as much as possible diverted from melancholy thoughts or fixed ideas. The *males* are, for the most part, in the fine seasons, occupied in the park and in the yards with gardening, digging, removing weeds, cleaving wood, pumping water, &c. There are, besides, special working shops for tailors, shoemakers, joiners, and saddlers; some occupy themselves with copying handwriting, painting, drawing, bookbinding, &c. In the winter, they assist within the house in the kitchen, in the forge, &c. The occupations of the *females* are spinning, weaving, darning, knitting, brewing, &c. All the works are of course superintended; and the rewards for diligence consist in a better fare, in praises, &c. Excursions are also often made to a larger park than that belonging to the hospital, a quarter of a mile distant, where the patients are refreshed with fruits, regaled with music, cigars, &c.

As to *pharmaceutical* remedies, Dr. G. keeps the just middle path between those who, with Heinroth and his (few) followers, consider the mind as alone suffering in the insane, and those who derive every species of lunacy from bodily disease. Both physical and moral treatment are considered necessary, and sometimes more of the one and sometimes of the other.

To this charitable institution also belongs another, for the relief and support of old decrepit people, unable to procure their sustenance. The building for these is entirely separated from the hospital; yet often incurable and quiet patients, and epileptic ones, are removed to it from the madhouse.

From the public hospitals we pass to *other charities for the poor*, of which we will only give a short account.

It was not before the year 1799 that the manner of taking care of the needy and poor in Denmark was founded upon sound and right principles. There are now twelve overseers of the poor for the twelve counties of Copenhagen; there is likewise a royal board of overseers, which consists of a president and four members, all of whom receive an annual yet small salary. The twelve overseers receive no salary. The total number of poor taken care of is about ten thousand; a number which readily explains the enormous debt, increasing with every year, which the board has incurred. The receipts are derived from funds supplied by the magistrates, from many legacies, from private beneficence, and from heavy taxes laid upon the inhabitants; but the expenses surpass by far the receipts, insomuch that the expenditure exceeds the income by no less a sum than 150,000 dollars, (15,000*l.*;) and the whole amount of the debt now is above a million and a half dollars. From this fund the poor and indigent are relieved by alms, good and free lodging, by being furnished with work and tools, by being taken care of when afflicted by diseases, and by getting their children educated at public expense. Public begging is on that account strictly prohibited, and every beggar is immediately arrested.

The metropolis is, as before mentioned, divided into twelve counties, and each has a physician appointed for the benefit of the poor in it. These either call on the physician or are visited by him, and receive medical attendance and medicine gratuitously.

Amongst the many charitable institutions, poorhouses, &c. for the indigent, the following deserve particular notice.

1. *The Almshouse in the General Hospital.* This has been already mentioned.

2. *Wartou, or the Hospital of the Holy Ghost.* This was founded in 1475, for the relief of poor old people, who here receive free lodging, bed, firing, a small weekly present, and free medical aid and medicine. There are at present in this institution 58 males and 358 females. It is not exclusively for the inhabitants of the metropolis, but also for others. Of the beds, 210 are private, and are disposed of by the proprietors. The right of founding such a bed is sold by the Board for the poor. To this charity-house a church is attached.

3. *The Hospital of Abel Cathrine.* This was founded in 1675, by *Abel Cathrine*, who was of the ancient noble family of *Van der Wisch*, and first lady of the household to the Queen Dowager Sophia Amelia. Twenty-three aged females are supported here by the bounty of the foundress, each of them occupying a small room, to which is attached a kitchen and pantry, and enjoying a weekly allowance of one and a half Danish dollar (2*s.* 6*d.*)

4. *St. John's House and Almshouse.* This has been already mentioned.

The following charitable institutions and poorhouses, destined for the relief and support of poor widows and other indigent people, not belonging to the lowest classes, are not under the inspection of the royal board of the poor, but are partly under that of the magistrates, partly under that of private persons according to the prescription of the founders.

5. *Harboe's Cloister.* This was founded in 1711 by the Dowager Lady Christiane Harboe, for the assistance and relief of twelve widow ladies of a certain rank. They have free lodging, and besides an annual allowance of money. The directress of this institution has the rank of prioress.

6. *Peterson's Cloister.* This foundation originates in the humanity of Albrecht and Sebastian Peterson; two brothers who were silk-mercers, and resided on the same spot where this establishment is now erected. On the wall over the windows of the second story is inscribed: "Monumentum pietatis Petersenianæ." Sixteen unmarried ladies reside here, enjoying two handsome rooms, kitchen, and cellar, with a yearly pension of about fifteen pounds sterling.

7. *Budolph's Cloister.* This was founded by a student in 1725, for the free residence of eight ladies.

8. *Paul Fechtel's Hospital.* This was endowed by Paul Fechtel, who was master of the mint in the reign of King Frederick the II. Fourteen old and infirm females have free lodging, and a weekly stipend of about one shilling English.

9. *Princess Charlotte's Institution.* This was founded by the daughter of King Frederick the IV., for the support of poor and destitute females of all ranks. The charity is divided into five classes. The first class is for the education of female children of the nobility; the second for daughters of gentlemen, who have held high situations under the government, and the three other classes are for respectable females of inferior rank.

10. *Stampe's Legacy.* This, amounting to about 8000*l.* was bequeathed for the education of poor children and for the assistance of old and infirm widows.

11. *Trösten's Bolig*, (The lodging of Consolation,) was founded by the late Admiral Winterfield. The object of this charity is the relief of worthy indigent widows, in order to afford them lodging at a small expense.

12. *Hans Peter Hofod's Legacy.* This gentleman founded a charity in 1812, for the relief of indigent seamen, their widows, and children, and endowed it with a bequest of about 20,000*l.*

13. *Naval Charity.* This noble charity owes its origin to the late Commodore Solling. It was founded in 1827, and affords relief at the present moment to thirty seamen, who by age, wounds, or infirmities, have a claim on this institution. It is entirely supported by voluntary contributions, and is under the immediate direction of ten Directors.

14. *Meyer's Memory.* This is a large institution for the support of persons of the Jewish religion, who here get free lodging.

15. *The united Charitable Society.* This is a very important institution, founded in 1788, for the purpose of aiding industrious and enterprising persons, who by misfortunes have become poor, by lending them money without interest, by which they may procure tools, &c. necessary in their profession. The Society, which at the present moment consists of 1,791 members, supports also poor members or their destitute widows, by annuities and other gifts. It is supported by legacies and by the publication of a weekly paper, "*Borgervennen*," (The Friend of the Citizen,) which has already existed forty years.

16. *The Female Charitable Society.* This was founded in 1825, and is restricted to females. It distributes every year, on the day of its foundation, premiums to female servants who have been long in service and conducted themselves faithfully and industriously. It supports a school for the daughters of poor parents.

17. *The Sisterly Charitable Society.* This is a similar charity, founded in 1792; it supports a school for daughters of destitute parents.

18. *The United Charity.* This Society was founded in 1832, for the support of poor and indigent persons in general. The members pay an exceedingly small sum, (only 4*s*. annually,) but their number compensates for this. Poor people are by this charity furnished with bread, firing, clothes, work, and their children with school-instruction.

19. *Wilhelmine's Institution.* This was founded by private subscriptions in 1828, in memory of the Princess Wilhelmine's nuptials. From the interests of the capital, on the 11th of May every year, a dowry of 500 dollars (50*l.*) is given to a daughter of a poor man in a royal office, and 200 dollars (20*l.*) to two craftsmen, in order to enable their settling.

20. *Caroline's Cloister.* This was also founded by private subscriptions, in 1829, to the memory of Princess Caroline's marriage. (Both princesses are the daughters of the present beloved king, and were married to princes of the royal house.) It is destined to afford free lodging and firing to a number of destitute widows and daughters, and to support them with money.

21. *The Royal Orphan-House.* This was founded by King Frederick the IV., in 1727, for the education of orphans or children (boys,) left by their parents. Besides the seventy-four orphans, who are educated in the house, many other children enjoy here gratuitous instruction, and are taught some handicraft. The institution takes also care of the education of many children in the provinces; at present 234 children may be reckoned to be the object of its care.

22. *The Royal Education-house*, ("Ortostringshuset.") This was founded in 1753, for the education of poor boys, who are here brought up to professions. The information given here is more extensive than is usual in the free schools, and no boy is admitted into the charity without being already imbued with a certain degree of elementary knowledge.

23. *The Deaf and Dumb Institution.* This was founded in 1817. The present king has bestowed on this noble establishment a fine building, where seventy boys and fifty girls are educated, clothed, and have every possible comfort. The first instructor, Mr. Schow, teaches religion, six other teachers different sciences, and two females are employed for the instruction of the girls. At a certain age boys educated here are instructed by teachers, who are deaf and dumb, in the profession of tailors, shoemakers, and weavers.

Mr. Amsel Meyer, who has bequeathed so many legacies to the different public charities of Denmark, left to this institution 4000*l.*, and every county in Denmark sending a child to this infirmary is subject to an annual payment of about 10*l.* derived from the poor-rates. The greater number of children who have been educated here are now following useful occupations.

24. *The Asylum and School for the Blind.* This was founded, 1811, by a private society of female free-masons, ("Kiædeselskabet,") and is still supported by it, by the king, and by various legacies. Twenty children are here instructed in religion, arithmetic, history, geography, natural history, and music; the females are taught spinning and knitting. To the school belongs, since 1825, a workhouse, into which the blind, after having perfected themselves in some handicraft, can be received, and where they may earn the fruits of their industry, have free lodging, &c.

25. *The Royal Humane Society.* This was established 1798, for the recovery of persons apparently drowned or dead. The Society has different receiving houses in the metropolis, all of which are supplied with excellent apparatus, and designated by conspicuous boards announcing their object. The Society distributes premiums to persons who have saved others from drowning, or exerted themselves in their recovery.

26. *Infant Schools.* There have been lately established, by private subscriptions, several asylums of this kind in different towns, where children between three and five years, who cannot be taken care of by their poor parents during their daily labours, are brought under inspection, and at the same time get such instruction as accords with their age.

Of late also a *Society for the bettering of criminals, and particularly of young delinquents*, has been established.

